# Supplementary material for: Transcriptome and Metabolome Analyses Reveal Sugar and Acid Accumulation during Apricot Fruit Development
Source: Int J Mol Sci. 2023 Nov 30;24(23):16992. doi: 10.3390/ijms242316992 (PMC10707722; doi:10.3390/ijms242316992)
Supplement: Supplementary file 1 [file ijms-24-16992-s001.zip › supplementary figures.pdf]

Supplementary Data For

Transcriptome and Metabolome Analyses Reveal Sugar and Acid Accumulation during Apricot Fruit Development

Ningning Gou [1,2,3,4], Chen Chen [1,3,4], Mengzhen Huang [1,3,4], Yujing Zhang [1,3,4], Haikun Bai [1,3,4], Hui Li [1,3,4], Lin Wang [1,3,4] and Tana Wuyun [1,3,4,*]

1 State Key Laboratory of Tree Genetics and Breeding, Research Institute of Non-Timber Forestry, Chinese Academy of Forestry, Zhengzhou 450003, China; lemonn@caf.ac.cn (N.G.); chenchenbo@caf.ac.cn (C.C.); mengzhen4524@163.com (M.H.); zhangyujing@caf.ac.cn (Y.Z.); bhk1994@163.com (H.B.); lihui19971204@163.com (H.L.); wanglin1815@163.com (L.W.)
2 College of Forestry, Nanjing Forestry University, Nanjing 210037, China
3 Kernel-Apricot Engineering and Technology Research Center of State Forestry and Grassland Administration, Zhengzhou 450003, China
4 Key Laboratory of Non-Timber Forest Germplasm Enhancement and Utilization of National Forestry and Grassland Administration, Zhengzhou 450003, China
* Correspondence: wuyuntana@caf.ac.cn

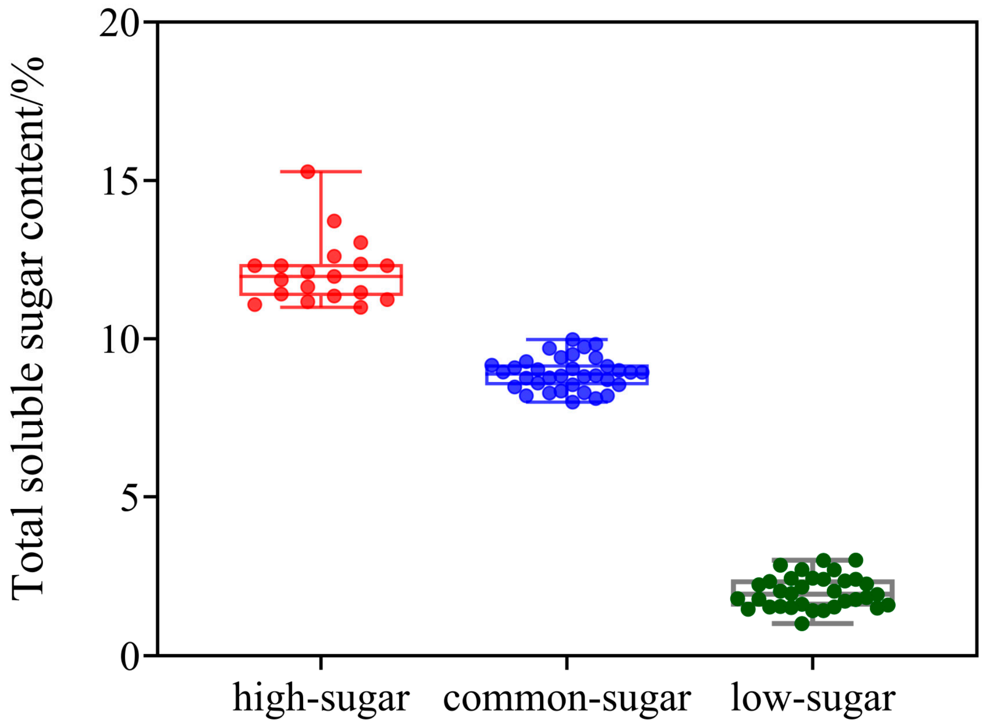

Supplementary FigureS1. Total soluble sugar content in different apricot fruits.

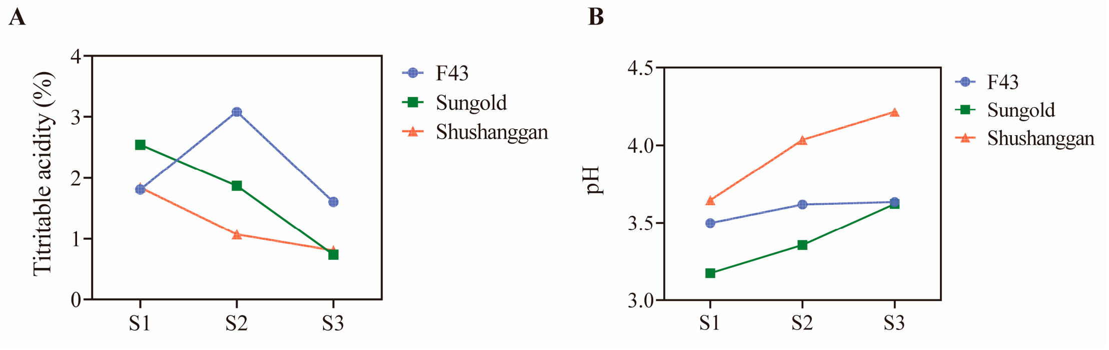

Supplementary FigureS2. Titritable acidity content and pH in different apricot fruits.

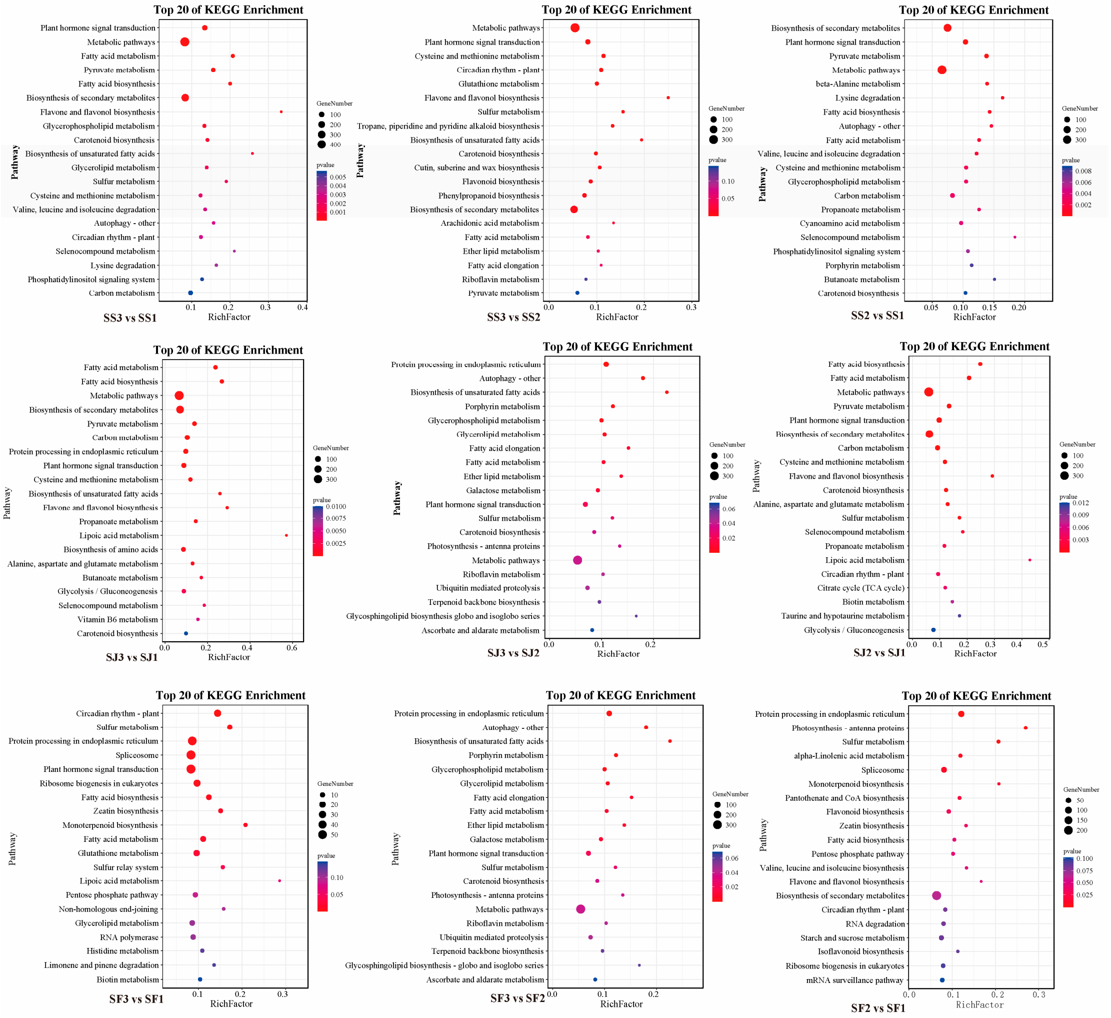

Supplementary FigureS3. DEmRNAs KEGG enrichment distribution map during fruit development in ‘shushanggan’, ‘sungold‘, and ‘F43’ during fruit development.

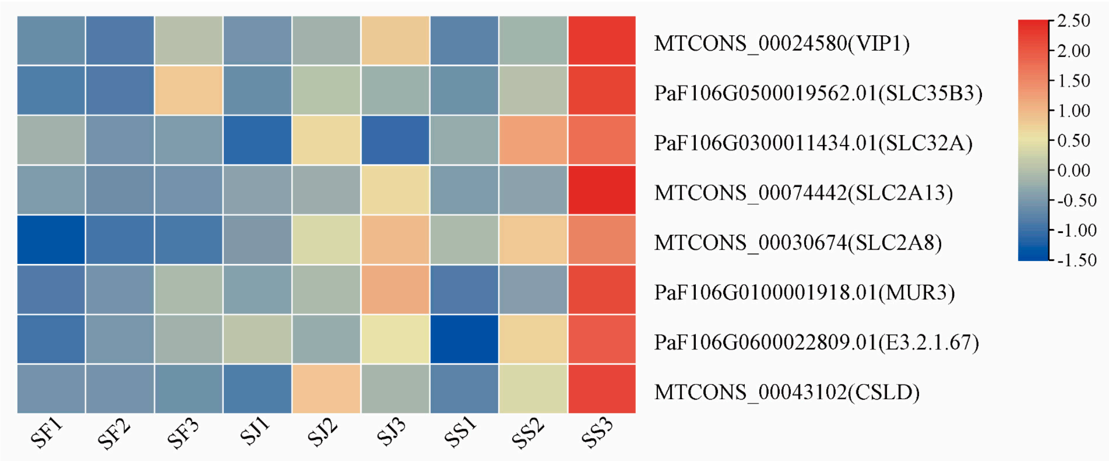

Supplementary FigureS4. Heat maps showing the expressions of eight hub DEGs in the MEbrown module of co-expression networks
